# Editorial: Metabolic regulation of stem cell fate

**DOI:** 10.3389/fcell.2025.1746636

**Published:** 2025-11-28

**Authors:** Rafael Sênos Demarco, Andrés Caicedo, Ana Caroline P. Gandara

**Affiliations:** 1 Biology Department, University of Louisville, Louisville, KY, United States; 2 Colegio de Ciencias de la Salud, Escuela de Medicina, Universidad San Francisco de Quito, Quito, Ecuador; 3 Instituto de Investigaciones en Biomedicina iBioMed, Universidad San Francisco de Quito, Quito, Ecuador; 4 USFQ Space Front, Universidad San Francisco de Quito, Quito, Ecuador; 5 Homeos Health Research, Quito, Ecuador; 6 Department of Genetics, University of Wisconsin-Madison, Madison, WI, United States; 7 Morgridge Institute for Research, Madison, WI, United States

**Keywords:** stem cell, metabolism, tissue-resident stem cell, mesenchymal stem cell (MSC), neural stem and progenitor cells/NPCs, hematopoietic stem and progenitor cells (HSPCs)

Stem cells have the crucial role to generate and maintain tissues and organs throughout life. These progenitor cells undergo asymmetric division to both self-renewal to maintain the stem cell pool, as well as to differentiate in order to generate specialized cells. In adults, stem cells reside in specific microenvironments, known as “niches,” that provide both structural and signaling cues for their maintenance and behavior. Systemic factors, such as the nutritional status of the organism, can also influence stem cell behavior either directly or through the modulation of niche function (Jones and Rando, 2011; Oh et al., 2014; Schofield, 1978; Voog and Jones, 2010; Drummond-Barbosa, 2019).

Recently, the role of metabolism has emerged as important for the regulation of stem cell behavior (Chandel et al., 2016; Clemot et al., 2020; Senos Demarco et al., 2020). From biases in the utilization of carbon sources to the overall state of cellular metabolism, metabolic processes have been shown to significantly contribute to the decisions stem cells undergo with regards to their proliferative, maintenance and differentiation capacity. Therefore, an emerging model suggests that metabolic adjustments are not merely permissive, but rather instructive, in the control of stem cell fate (Labusca et al., 2025).

This Research Topic, entitled *Metabolic Regulation of Stem Cell Fate,* adds to the growing knowledge of how metabolic organelles and metabolites can influence stem cell behavior. Mesenchymal stem cells (MSCs) have multifaceted roles in regenerative medicine. Not only can MSCs generate several different specialized cell lineages, but they also participate in cytokine secretion and immunomodulation, particularly at injury sites (Pittenger et al., 2019). The perspective by Jaraba-Álvarez et al. explored how, in MSCs, a metabolic adaptation happens in response to hypoxia to enhance cellular survival and activity. Focusing on the role MSCs play in tissue regeneration, the authors built the argument that low oxygen levels would be optimal when culturing MSCs for therapeutics. Hypoxia influenced several cellular processes, including the activation of the hypoxia-inducible factor 1-alpha (HIF1α) response and shifts in mitochondrial respiration and in intermediate metabolites. Hypoxia pre-conditioning improved HSC homing to injury sites and secretion of exosomes and extracellular vesicles; all of which can contribute to tissue repair and regeneration.

Due to its accessibility and lineage potential, adipose-derived MSCs (AD-MSCs) can be used as a source for cardiac regeneration. Farag et al. performed untargeted metabolomic profiling to identify specific metabolic pathways that were activated during MSC-to-cardiomyocyte differentiation. Interestingly, their studies revealed that AD-MSCs harvested from different sources have distinct metabolic profiles while undergoing cardiomyocyte differentiation: peri-ovarian AD-MSCs displayed a much broader metabolic reprogramming with enhanced flexibility and energy efficiency than peri-renal AD-MSCs. Their results highlight the importance of understanding, from a metabolic perspective, how different stem cell pools may be more or less suitable for cardiac regenerative approaches.

As key players in cellular metabolism, mitochondria have emerged as important regulators of stem cell behavior in several tissues (Labusca et al., 2025). In neural stem and progenitor cells (NPSCs), Bustamante-Barrientos et al. uncovered the role of mitochondria-derived reactive oxygen species (ROS) in response to the chemotherapy agent cisplatin. Lower (non-cytotoxic) cisplatin concentrations disrupted mitochondrial activity and increased ROS production, affecting NPSC homeostasis by promoting differentiation at the expense of proliferation and self-renewal. Interestingly, antioxidant treatment could rescue the differentiation bias but not defects in proliferation and self-renewal. These results demonstrate the lasting effects of cisplatin-caused disruption in mitochondrial homeostasis in NPSCs, which could better inform future strategies to prevent brain damage during chemotherapy.

Mitochondrial homeostasis is also important for proper hematopoiesis. Batabyal et al. showed that disruption of mitochondrial AAA+ in differentiated *Drosophila* blood cells (hemocytes) caused non-autonomous changes to hematopoiesis, including an expanded progenitor cell niche. Reduction of ROS levels caused by the disruption of mitochondrial homeostasis in hemocytes restored progenitor cell niche size and differentiation potential, showcasing how mitochondrial metabolism in differentiated hemocytes can signal back to control the activity of the progenitor cell niche.

Together, the articles in this Research Topic help advance our understanding of how metabolism can influence stem cell fate. By providing evidence that mitochondrial metabolites can act as signaling molecules to influence mesenchymal, neural and hematopoietic stem and progenitor cells, these studies highlight the importance of understanding the metabolic profiles of these cells in order to further the development of stem cell-based regenerative therapies.
